# A qualitative study of the impact of endometriosis on male partners

**DOI:** 10.1093/humrep/dex221

**Published:** 2017-06-29

**Authors:** L. Culley, C. Law, N. Hudson, H. Mitchell, E. Denny, N. Raine-Fenning

**Affiliations:** 1 School of Applied Social Sciences, Faculty of Health and Life Sciences, The Gateway, De Montfort University, Hawthorn Building, Leicester LE1 9BH, UK; 2 Faculty of Health, Education and Life Sciences, Birmingham City University, Westbourne Road, Edgbaston, Birmingham B15 3TN, UK; 3 Nurture Fertility, East Midlands Fertility Centre, 25 Business Park, Bostocks Lane, Sandiacre, Nottingham NG10 5QS, UK; 4 Division of Obstetrics and Gynaecology, School of Clinical Sciences, University of Nottingham, University Park, Nottingham NG7 2RD, UK

**Keywords:** endometriosis, psychosocial care, quality of life, couple, male partner, qualitative research, men, intimacy, well-being

## Abstract

**STUDY QUESTION:**

What is the impact of endometriosis on male partners of women with the condition?

**SUMMARY ANSWER:**

Endometriosis significantly impacts men across several life domains and can negatively impact emotional well-being.

**WHAT IS KNOWN ALREADY:**

Endometriosis has been shown to negatively impact women's quality of life and may strain intimate relationships. Little is known about the impact on male partners.

**STUDY DESIGN, SIZE, DURATION:**

The ENDOPART study was a cross-sectional, qualitative study of 22 women with endometriosis and their male partners (*n* = 44) in the UK (2012–2013).

**PARTICIPANTS/MATERIALS, SETTING, METHODS:**

Inclusion criteria: laparoscopic diagnosis of endometriosis; the presence of symptoms for at least a year; partners living together. Data were collected via face to face, semi structured interviews with partners interviewed separately. Data were analysed thematically, assisted by NVivo 10.

**MAIN RESULTS AND THE ROLE OF CHANCE:**

Men reported that endometriosis affected many life domains including sex and intimacy, planning for and having children, working lives and household income. It also required them to take on additional support tasks and roles. Endometriosis also had an impact on men's emotions, with responses including helplessness, frustration, worry and anger. The absence of professional or wider societal recognition of the impact on male partners, and a lack of support available to men, results in male partners having a marginalized status in endometriosis care.

**LIMITATIONS REASONS FOR CAUTION:**

Self-selection of participants may have resulted in a sample representing those with more severe symptoms. Couples included are in effect ‘survivors’ in relationship terms, therefore, findings may underestimate the contribution of endometriosis to relationship breakdown.

**WIDER IMPLICATIONS OF THE FINDINGS:**

The study extends knowledge about the impact of endometriosis on relationships, which thus far has been drawn largely from studies with women, by providing new insights about how this condition affects male partners. Healthcare practitioners need to take a more couple-centred, biopsychosocial approach toward the treatment of endometriosis, inclusive of partners and relationship issues. The findings demonstrate a need for information and support resources aimed at partners and couples.

**STUDY FUNDING/COMPETING INTEREST(S):**

This study was funded by the Economic and Social Research Council (reference ES/J003662/1). The authors have no conflicts of interest.

## Introduction

Endometriosis is a chronic gynaecological condition in which endometrial-like tissue is present outside the uterus, inducing a local inflammatory response (Kennedy *et al.*, 2005; De Nardi and Ferrari, 2011). Prevalence is difficult to estimate, but it is generally thought to affect between 2 and 10% of women of reproductive age (Eskanazi and Warner, 1997). Common symptoms are chronic pelvic pain, fatigue, congestive dysmenorrhoea, deep dyspareunia and subfertility (Lemaire, 2004; Meuleman *et al.*, 2009; De Nardi and Ferrari, 2011). Some women will experience severe symptoms while others will be asymptomatic or only experience mild symptoms. A range of treatments are available to relieve symptoms, including analgesics, hormonal treatments and surgery, with varying degrees of success (European Society of Human Reproduction and Embryology (ESHRE), 2013), though there is no definitive cure. Despite treatment a substantial proportion of women still suffer pain of varying types and levels of intensity (De Graaff *et al.*, 2013).

There is a growing body of research (both quantitative and qualitative) which documents the negative impact of endometriosis on the quality of life of women (Gao *et al.*, 2006; Jia *et al.*, 2012; Culley *et al.*, 2013a; Young *et al.*, 2015). Studies with women have reported a negative effect on sexual function (Pluchino *et al.*, 2016) and strain on intimate relationships, which in some cases contributed to relationship breakdown (Cox *et al.*, 2003; Jones *et al.*, 2004; Denny, 2004a,b; Huntington and Gilmour, 2005; Fagervold *et al.*, 2009), as well as identifying partners as the greatest source of support for women (Denny, 2004a; Denny and Mann, 2007). However, such studies have primarily focussed on women's accounts and the voices of male partners remain largely absent from the literature (Culley *et al.*, 2013a; Pluchino *et al.*, 2016). Exceptions include a study that explored the experiences of 16 male partners of women with endometriosis, describing men's emotional responses as mirroring the Kubler-Ross grieving process, including shock and denial, anger, anxiety, isolation and powerlessness, low mood and also acceptance and relationship growth (Fernandez *et al.*, 2006). These findings were confirmed by a study of 13 couples, reporting disruptions to day-to-day life and a significant impact on sexuality and intimate relatedness (Butt and Chesla, 2007). Quantitative studies have suggested that sexual functioning in male partners is not affected by endometriosis (De Graaff *et al.*, 2016). Qualitative studies offer a means to more fully explore partners’ perceptions of the multiple ways in which sex and intimacy may be affected.

A growing body of literature indicates significant psychosocial effects on partners of those with a range of chronic conditions (Baanders and Heijmans, 2007; Luttik *et al.*, 2009; Ervik *et al.*, 2013). Furthermore, research on conditions such as rheumatoid arthritis (Mann and Dieppe, 2006; Sterba *et al.*, 2008), cardiac disease (Mahrer-Imhof *et al.*, 2007), lupus (Fekete *et al.*, 2007) and cancer (Maughan *et al.*, 2002; Schulz and Schwarzer, 2004) indicates the significant impact of partner perceptions of the ‘ill’ person and the importance of partner support in coping with chronic conditions.

The 2013 ESHRE Guideline on The Management of Women with Endometriosis highlights the need to investigate the psychosocial impact of endometriosis on women and partners. The analysis which follows is drawn from the ENDOPART study which was designed to qualitatively investigate the impact of endometriosis on the well-being of women, men and couples (Culley *et al.*, 2013b). The paper argues that men are often marginalized in relation to endometriosis. There is a need for greater recognition of the impact on male partners and the development of additional couple orientated support.

## Materials and Methods

### Aims

The aims of the ENDOPART study were to explore the impact of endometriosis on women and their male partners; contribute to theory development in chronic illness; and improve the well-being of people living with endometriosis by providing an evidence base for improving couple support.

### Ethical approval

Approval was received from the host university and the East Midlands Leicester NHS Local Research Ethics Committee UK (reference 12/EM/0015). Participants were required to provide written consent.

### Study design

The ENDOPART study was a cross-sectional, qualitative study. The first phase comprised context-setting interviews with key informants (*n* = 11) including healthcare practitioners, patients and support group representatives, and a systematic literature review (Culley *et al.*, 2013a). Phase two of the study involved face to face, in depth, semi structured interviews with 22 women with endometriosis and their male partners (*n* = 44). Interviews lasted between 50 and 113 min (mean 78 min). Multiple recruitment routes were utilized. Inclusion criteria were laparoscopic diagnosis of endometriosis; the presence of symptoms for at least a year; and partners living together. The interview guide was devised based on themes identified in the literature review (Culley *et al.*, 2013a) and findings from context-setting interviews in phase one. Interview schedules covered the same topics for women and men. This guide was piloted and amendments made. Topics included symptom onset and the journey to diagnosis, understandings of the causes of endometriosis, the impact of endometriosis on everyday life and on relationships, experiences of healthcare, communication and support within relationships, external support and information, and feelings about the future. Women and their male partners were not interviewed together, but were interviewed separately and where possible simultaneously by different interviewers. Data saturation was achieved at 44 interviews. Sample characteristics are provided in Tables I and II. The interviews were audio recorded and transcribed verbatim.

**Table I:** Age and ethnicity of study participants (*n* = 44).

	Number of women	Number of men
Age		
25–30 years	8	8
31–40 years	11	9
41–50 years	3	3
51 years or older		2
Mean age	34.8	36.3
Ethnicity		
White British	14	13
South Asian	6	6
Other	2	3

**Table II:** Circumstances and recruitment routes for couples participating in the study (*N* = 22).

	Number of couples
Length of relationship at interview	
0–5 years	9
6–10 years	5
11–15 years	5
16–20 years	2
21 years or more	1
Mean	9.1
Length of time since women's onset of symptoms at interview	
0–5 years	4
6–10 years	7
11–15 years	4
16–20 years	3
21–25 years	1
26–30 years	2
31–35 years	0
36 years or more	1
Mean	13.6
Length of time since women's diagnosis at interview	
0–5 years	16
6–10 years	4
11–15 years	1
16–20 years	1
Mean	4.5
Recruitment to study	
Endometriosis UK	11
Secondary/tertiary clinics	5
Other support/information organizations	3
Word of mouth	3

### Analysis

Interview data were analysed thematically (Braun and Clarke, 2006). A coding framework was drafted by one researcher (C.L.), identifying codes from the literature review, interview guide and full data set; five researchers (L.C., N.H., H.M., E.D. and C.L.) reviewed this framework in relation to a sample of the data (12 interviews, or 27%) using a team analysis approach and amendments were made. A sample of data was then coded by two researchers (C.L. and H.M.), using the coding comparison function in the software package NVivo (software supplier: QSR International; see www.qsrinternational.com/nvivo-product) to measure inter-coder reliability: this reported a 98.4% agreement, with a Kappa coefficient of 0.41 (i.e. ‘fair to good’ agreement). This process enabled the refining of the coding framework: two researchers discussed all codes with a Kappa coefficient of <0.40 (i.e. ‘poor’ or ‘no’ agreement), and reviewed how codes or descriptors of codes had been interpreted differently; these were then amended to ensure clarity. The coding framework was then finalized, the entire data set coded, and an overarching thematic description of the data set was produced. Data pertaining to themes identified to be particularly significant at the couple level were then also analysed dyadically, taking each couple as a ‘unit of analysis’ and exploring contrasts and overlaps in partners’ accounts (Eisikovits and Koren, 2010; Hudson *et al.*, 2016). In order to focus analysis on the impact on male partners, further qualitative analysis was conducted in which themes from the original thematic analysis were reviewed and those compatible with the concept of ‘impact’ were identified. Data from male partners relevant to these themes were extracted and summaries of these data are presented below.

## Results

The findings presented here report key themes relating to the perspectives of male partners and are discussed under the following thematic headings: sex and intimacy; planning for and having children; men's working lives and household income; additional support tasks and roles; the emotional impact of endometriosis on male partners; lack of support; and positive impacts. Quotes from male partners are presented to illustrate the description of findings. To maintain anonymity within the couple unit (i.e. to prevent participants from identifying quotes from their partner) we have refrained from using descriptive labels, identifiers or pseudonyms. Unless necessary to provide contextual information, data from the female partners within the couple units have not been presented. Women's accounts are provided in Culley *et al.* (2013b) and Hudson *et al.* (2016).

### Sex and intimacy

The impact of endometriosis on sex and intimacy for couples was profound. In nearly half (*n* = 11) of the couples sex was reported to be ‘non-existent’ or ‘rare’, either at the time of the interview or in a recent phase, and others (*n* = 7) reported reduced frequency of sex. Men spoke at length about the impact of endometriosis on sexual relations with their partners, though they were less likely than women to report a significant loss of intimacy, closeness and affection. The impact on sex was not solely related to dyspareunia, reported as a symptom by 19 women, but also to women experiencing general fatigue, reduced sexual desire as a result of medication, low mood, the stress of trying to get pregnant, bleeding during and/or after sex, and women feeling generally unattractive and unfeminine.

Most men were aware of the potential for pain with intercourse which may make them hesitant regarding physical intimacy. While this was expressed in some form by many men, four men reported explicitly that this made them reluctant to approach their partner to initiate sex.

‘I wouldn't try because it would be like, it's going to be pain to you and the last thing I want to do is for her to be in pain.’

Men took two stances on whether a reduction in the amount of sex in the relationship was problematic: while a minority (*n* = 5) regarded the lack of sex as a very significant problem for them, 12 men explicitly stated that it was not problematic and that they accepted the situation. However, this acceptance was often couched this in terms of having ‘learned to live with it’ or alongside a suggestion that it would be ‘unreasonable’ or ‘selfish’ of them not to accept it. Conversely, three men appeared to fall into both categories and seemed comfortable in expressing these two seemingly conflicting positions: expressing acceptance and understanding while also acknowledging dissatisfaction and loss. Overall, within men's accounts there appeared to be a tension between men seeking to acknowledge dissatisfaction with the impact of endometriosis on sex and intimacy while also seeking to avoid appearing to ‘blame’ their female partner or being perceived as selfish or unreasonable.

‘I'd be a pretty shit husband if I was like ‘well this is rubbish isn't it’ … you've just got to take it on the chin really … if it's my needs or whatever, who gives a toss about that, let's, like I said, get her better. It's not about me.’

A minority of couples (*n* = 3), directly stated that the lack of sex led to tensions and arguments, and even for those who did not specifically disclose conflict, it was evident that some couples had not found alternative ways of expressing closeness. So, despite expressing a sense of resignation and acceptance, across the data set the impact on men was considerable.

### Planning for and having children

Eighteen out of 22 couples reported that endometriosis had in some way affected their plans with regard to having children. In half of these cases (*n* = 9), couples had experienced fertility problems and pursued fertility investigations and/or treatment, and in some cases IVF or adoption. The other nine couples described endometriosis affecting their decision-making about whether or when to have children and how many. For example, two couples had tried to conceive earlier than they would have otherwise out of concern that endometriosis might affect fertility; one had fewer children than they had initially desired; and one decided not to have children at all. Therefore, either actual or *anticipated* infertility was a significant issue for the majority of the couples. The emotional impact of this on women and men varied but few men experienced the same levels of anxiety and concern reported by women.

Amongst the nine men in couples who had not sought and/or undertaken investigations or treatment, a small minority (*n* = 3) described feelings of sadness, worry or shock at (actual or anticipated) difficulties conceiving. However, most felt that the emotional impact was greater for their partner. They appeared to experience considerably less anxiety and worry about anticipated infertility than women, and reported adopting a ‘wait and see’ attitude.

‘I know it affected [partner] worse because she thought the worst and that she wouldn't be able to have kids, whereas I am a bit more, didn't really worry about it until we needed to … we will cross that bridge when we come to it.’

However, amongst the nine men in couples who *had* sought and/or received medical fertility investigations or treatment, the emotional impact was far more pronounced than amongst the men in couples who had not (yet) sought investigations or treatment. Men described feelings of disappointment, stress, isolation, distress, trauma and envy, and for a minority these feelings were profound (*n* = 4). For these men, the impact of endometriosis on planning for and having children eclipsed other experiences relating to the condition.

‘Coming to terms with not having children of our own and the whole process of IVF, going through it, is really traumatic and for me that's been the most painful element of the whole process … I have never gone through the kind of extended period of profound misery and disappointment as I have with IVF.’

### Men's working lives and household income

The impact of endometriosis on men's working lives was far less significant than on women's working lives. However, several did describe endometriosis as affecting them in relation to employment in some way (*n* = 5). The condition made demands on men's time which affected their paid work, for example, accompanying their partners for consultations and undertaking extra childcare and housework when partners were unwell. A small proportion of men reported that there were specific times (such as surgery or medical crisis) when the challenges of managing paid work, supporting their partner (see below), and undertaking household tasks and childcare affected their productivity and concentration at work (*n* = 4). One man mentioned trying to find work closer to home and several men reported that they relied on the flexibility of self-employment or professional jobs that allowed them to take time out for appointments or when their partner had surgery (*n* = 5).

‘I guess it does [have an impact on my working life] … I guess it's emotional, not tiredness but … emotional burnout I guess. But I was able to work. But it would be more difficult because of what was happening with her.’

Both men and women noted the impact that endometriosis had on household income because of the loss or reduction of the women's income, and the additional costs associated with hospital treatment and/or IVF. Some men reported feeling the added pressure of providing a larger share of the household income as their partner's earnings were affected by her illness, while others spoke of how the female partner not working or working reduced hours had created a change in their relationship and for some this meant a reversion to traditional gender roles of male-breadwinner and female-dependant.

‘She wants to go out and get a job, she wants to earn money, she wants to earn her own money because at the moment if she wants anything she has to ask me for money. We have accidentally become a traditional 1950 s household, the man goes out to work all day and the little lady is at home!’

### Additional support tasks and roles

Endometriosis changed the roles that men occupied in relation to the nature and extent of support they provided within the relationship, in three areas. First, men provided support in relation to healthcare and treatment. This included attending consultations, discussing treatment options and helping to make decisions, helping women with self-management and providing care after surgery. Second, men provided support in relation to managing everyday life. Men took on additional tasks in managing the home and looking after children, both on a day-to-day basis and/or while female partners recovered from surgery. Participants also described additional practical support such as driving their partner to and from work, and contributing more financially. Third, men provided emotional support to their female partners. Men described their roles as involving caring, listening, understanding, ‘being there’, being available to talk things through and taking their partner's feelings and needs into consideration.

### The emotional impact of endometriosis on male partners

Most men reported that the research interview was the first time that they had been asked how they felt about endometriosis and the ways in which it affected them. While in general men had some initial difficulty in articulating how endometriosis had affected them, and some difficulty in naming and describing emotions, careful and sensitive probing revealed a range of impacts. A minority of men reported very little influence on them emotionally (*n* = 5). The majority reported experiencing strain, stress and/or distress, to some extent (*n* = 17). Overall, four main emotions were apparent throughout the data set: helplessness, frustration, worry and anger.

Some men described feeling helpless and powerless in response to their inability to alleviate their partner's symptoms. Frustration also characterized many men's responses: frustration that they could not relieve their partner's symptoms, especially pain, or that endometriosis prevented the couple having the life and the relationship they wanted to have.

‘You just feel helpless, try to do as much as you can for them like but, and try to do the best you can, but there's not much that you can do. Nothing you do will relieve their pain.’

‘You almost kind of feel impotent in your actions because there is nothing you can do to make her better.’

Men also commonly described experiencing considerable worry about their partner's well-being, anxiety around diagnosis and surgery, and concern about possible infertility and about the long-term effects of treatment.

‘I do worry how it will affect her health and if it's going to spread and then how it's going to spread, what can we do to contain it.’

Some men also described feelings of anger, which were directed in large part at the healthcare received by partners. Several men described feeling frustrated and critical of medical management and healthcare practitioners, and expressed their dissatisfaction with the quality and timeliness of their partners’ diagnosis, treatment and care. Men were more likely than women to perceive the goal of endometriosis management as identifying the cause and ‘sorting out’ the problem and consequently felt frustration and sometimes anger when medical management failed to achieve this mechanical ‘fix’.

Men reported finding it difficult to cope with the impact of endometriosis on their partner's emotions. The majority of women reported that the symptoms and treatment of endometriosis resulted in them feeling low, depressed, tearful and/or irritable and angry and men described feeling overwhelmed and aggrieved at the ways in which these emotions affected the couple dynamics.

‘[You are] the verbal punch bag so to speak, just get it all because she's so down and depressed or wound up over it and in pain.’

For a minority of men (*n* = 4), the impact of endometriosis was described as all-encompassing, affecting them on a daily basis to the extent that some felt they ‘had no life’.

‘You cannot say a specific thing, it's all the ways, whatever you want to do, whatever you think, endometriosis is stopping you. So there's no specific thing actually, it's like everything … it's just locking you up.’

However, few men reported that they discussed with their partners the ways in which endometriosis affected them emotionally. They expressed the view that their own feelings were insignificant in comparison to their partner's, and so were dismissive of them within the relationship. Some men talked about how they conceal their own emotions and put on a brave face in order to protect or shield their partner, and try to ‘stay strong’ and demonstrate positivity.

‘I don't really tend to show a lot of emotion … if she breaks down and she sees me sort of faltering, it's not going to give her much support. So I guess the old male stereotype kicks in and you have to be seen to be the stronger one.’

### Lack of support

Men were asked what support they receive from their partner, and from others outside the relationship, and what additional support they would like to receive. Some men stated that they did not need any support (*n* = 5). However, the majority identified a lack of support for male partners and felt more support was needed—either for themselves directly or for male partners more generally (*n* = 17).

Although many men (*n* = 8) felt that their partner was supportive towards them, several seemed reluctant to voice to their partner how endometriosis affected them for fear of appearing selfish. They described how they felt it would be unreasonable to expect their partner to more fully consider the impact on them and provide support, when these women were dealing with pain and other symptoms of endometriosis.

‘I can't look for support when she was in pain; because I guess then it's my role to support her.’

Outside the relationship, few men (*n* = 6) received support from friends, family or healthcare professionals—although those who did said that they had benefitted from this. Men perceived that family and friends, and indeed wider society, lacked a sufficient awareness and understanding of endometriosis and its effects.

Men suggested several forms of support they would welcome including opportunities to communicate with people outside the relationship who understood the condition; information about endometriosis and its effects; and reassurance, emotional support and the opportunity to offload emotions.

‘Everybody needs to talk freely [away] from their partner … you need to talk it through with someone else. It is kind of a thing, people don't really think about the guys. But it is, an awful lot of emphasis is put on the lady, but the guy has to deal with an awful lot as well. And especially because it's not very well known, it's hard to find someone to talk to who understands.’

Men reported that few healthcare practitioners recognized the ways in which endometriosis affected them.

‘It has been hard because, not that I would want it to be the other way, the focus clearly has to be on the woman for obvious reasons, she's the one in pain and discomfort … but you do at times think ‘what about me, no-one's asked me how I'm feeling’. There are times when you think the bloke doesn't get a look in.’

### Positive impacts

Although men reported several negative and challenging ways in which endometriosis affected them, they also identified positive impacts of the condition. Men described how living alongside the condition, and developing their approach to supporting their partner, had enabled them to become a more sympathetic person and a better partner, capable of listening and offering support. Others described how their experiences had enabled them and their partner to become closer and had strengthened their relationship.

## Discussion

Just as there is huge variance in women's experiences of endometriosis (ESHRE, 2013), the ways in which partners are affected also varies considerably. Nonetheless, this study provides evidence that endometriosis can have a significant impact on male partners, affecting many life domains including sex and intimacy, planning for and having children, working lives and household income and support roles, and also has a substantial influence on men's emotions. The findings also demonstrate the absence of sufficient support to help men deal with these impacts, resulting in men feeling isolated and unsupported; therefore, the study suggests that male partners are marginalized in relation to endometriosis. Indeed men commented that the interview was the first time they had been asked about and had the chance to articulate their experiences. Men reported that few healthcare practitioners recognized the impact endometriosis had on them, as their focus is, understandably, on the female partner (Maughan *et al.*, 2002). Within endometriosis support provision, we found little information or support aimed at partners, and the men in the study confirmed this absence. Within relationships, most men refrained from discussing their feelings with partners to any great extent, and support from family or friends was minimal. Thus, male partners and their needs appear to be marginalized at several levels: healthcare, information and support, within relationships and within wider society and social life.

The ways in which endometriosis impacts on male partners, and how men in turn then respond to these impacts, appear to be influenced by cultural expectations associated with masculinity. While notions of masculinity are complex and multifaceted, at the general level masculine roles and scripts encourage men to appear strong, stoical, rational, unemotional and assertive (Connell, 1995). A commitment to appearing strong and stoical may function to reinforce the marginalization men experience, as they are reluctant to articulate their experiences and feelings or to seek support. It may also shape the behaviour of healthcare practitioners (Maughan *et al.*, 2002). Thus, marginalization is likely to be compounded by issues of gender and masculinity.

The impacts on men may have a bearing on the health and well-being of men and may in turn impair relationships and the ability of men to fully support their partners. For most people with chronic conditions in relationships, the behaviours and beliefs of partners are of considerable importance in how the condition is experienced and managed (Henry *et al.*, 2013; Pereira *et al.*, 2016).

These findings have implications for the delivery of healthcare and for information and support. Due to the physical, emotional and social needs associated with endometriosis, the importance of patient-centeredness in endometriosis care has been highlighted previously, and aspects of care relating to partners and relationship issues are included within the ENDOCARE measure (Dancet *et al.*, 2011). The ENDOPART study further reinforces the need for a biopsychosocial approach toward the treatment of endometriosis, inclusive of partners and relationship issues, and to offer not only a more patient-centred approach but, beyond this, a couple-centred approach. The adoption of such an approach at an organizational level in healthcare delivery, aided by use of the ENDOCARE tool, should be considered. Relevant professional bodies should consider incorporating information about the psychosocial impact of endometriosis on women, partners and couples in professional training. Furthermore, wider societal awareness regarding the psychosocial impact of endometriosis on women, partners and couples is necessary to enhance social support for this enigmatic and little-known condition, and endometriosis support groups can facilitate this.

The findings also show a need for information and support resources aimed at partners and couples, and for further work addressing the relational effects of the condition and how couples (including non-heterosexual couples) can cope better (see Culley *et al.*, 2013b for further recommendations from the study). Based directly on the findings of the ENDOPART study, De Montfort University and leading UK charity Endometriosis UK (www.endometriosis-uk.org) have jointly produced several new materials, including online information, an educational intervention deliverable by support groups, and a film featuring real-life couples and advice from a clinical psychologist, to be launched 2017.

### Limitations

The self-selection of participants to the study may have resulted in a sample representing those with more severe symptoms. The couples sampled are in effect ‘survivors’ in relationship terms; therefore, these findings may underestimate the ways in which endometriosis contributes to relationship breakdown. The study focused on the experiences of heterosexual couples.

## Conclusion

The ENDOPART study was the first UK-based qualitative study to explore the impact of endometriosis on male partners of women with the condition, and provides evidence that the impact on partners can be profound. Life domains including sex and intimacy, planning for and having children, and working lives and household income were affected, and men reported undertaking new and varied support tasks. Importantly, despite men identifying some positive aspects of the condition, the qualitative approach uncovered a substantial impact on the emotional well-being of men. However, men's experiences appear to be largely unacknowledged and men are marginalized in multiple ways. The study demonstrates a need for healthcare services, as well as support and information resources, to be offered within a holistic, biopsychosocial model, which includes a consideration of the needs and experiences of women's partners and of couple relationships.
